# CD8+ T-Cell Interleukin-7 Receptor Alpha Expression as a Potential Indicator of Disease Status in HIV-Infected Children

**DOI:** 10.1371/journal.pone.0003986

**Published:** 2008-12-19

**Authors:** Tanvi S. Sharma, Jane Hughes, Amarylis Murillo, Joanne Riley, Andreia Soares, Francesca Little, Charles D. Mitchell, Willem A. Hanekom

**Affiliations:** 1 Division of Pediatric Infectious Diseases, Children's Hospital Boston, Harvard Medical School, Boston, Massachusetts, United States of America; 2 South African Tuberculosis Vaccine Initiative, School of Child and Adolescent Health, Cape Town, South Africa; 3 Division of Pediatric Infectious Diseases, Miller School of Medicine, University of Miami, Miami, Florida, United States of America; 4 Department of Statistical Sciences, University of Cape Town, Cape Town, South Africa; New York University School of Medicine, United States of America

## Abstract

**Background:**

Initiation and modification of antiretroviral therapy in HIV-infected children depend on viral load and CD4+ T-cell count. However, these surrogates have limitations, and complementary immunological markers to assess therapeutic response are needed. Our aim was to evaluate CD8+ T-cell expression of CD127 as a marker of disease status in HIV-infected children, based on adult data suggesting its usefulness. We hypothesized that CD127 expression on CD8+ T-cells is lower in children with more advanced disease.

**Methods:**

In a cross-sectional evaluation, we used flow cytometry to measure CD127+ expression on CD8+ T-cells in whole blood from HIV-infected children with varying disease status. This was compared with expression of CD38 on this subset, currently used in clinical practice as a marker of disease status.

**Results:**

51 HIV-infected children were enrolled. There was a strong positive correlation between CD127 expression on CD8+ T-cells and CD4+ T-cell count, and height and weight z-scores, and a strong negative correlation between CD127 expression and viral load. In contrast, we found no association between CD38 expression and these disease status markers.

**Conclusions:**

CD8+ T-cell CD127 expression is significantly higher in children with better HIV disease control, and may have a role as an immunologic indicator of disease status. Longitudinal studies are needed to determine the utility of this marker as a potential indicator of HIV disease progression.

## Introduction

Laboratory assessment of HIV disease status is indispensable for managing antiretroviral therapy in infected children. Initiation and modification of antiretroviral therapy depend, to a large extent, on plasma viral load and peripheral blood CD4+ T lymphocyte count. Although these surrogates for monitoring disease status are widely used, there are limitations. For example, the kinetics and extent of antiretroviral-mediated CD4+ T cell recovery may show great variation from child to child [1]. Similarly, in many settings CD4+ T-cell counts may correlate poorly with viral replication [1].

There has been a search for complementary immunological markers to assess or predict response to therapy. In the pediatric setting, most reports have been generated by relatively small studies. For example, T-cell expression of HLA-DR, CD38 and CD95 has been correlated with a poor response to therapy [2], [3]. Functional characteristics of T-cells have also been evaluated. Predominant production of interleukin (IL)-2 by HIV-specific T-cells may be associated with a favorable response to antiretrovirals, in contrast to the poorer outcome when IFN-γ production is more prevalent [4]–[6]. Two studies in the United States of novel immunological correlates of response to therapy have involved large numbers of infants and children [5], [6]. Both studies found that a decrease in CD8+ T-cell percentage, and in CD8+CD38+HLA-DR+ percentage, is associated with a favorable response to therapy. The authors suggested introduction of such markers into clinical pediatric practice.

More recently, numerous studies have indicated that T-cell expression of IL-7 receptor alpha (IL-7Rα or CD127) may be a predictor of clinical HIV disease status in adults [7]–[15]. These studies show that CD127 expression on T-cells is lower in HIV-infected individuals compared with uninfected controls, and that this expression correlates inversely with disease severity. Some reports have suggested that the marker may be useful for predicting response to therapy [7], [9], [10], whereas another has questioned its utility [14]. CD127 is the receptor for IL-7, a cytokine crucial for thymopoiesis, maintenance of CD8+ memory T-cells, and peripheral T-cell homeostasis in states of T-cell depletion [16]–[18]. Progressive HIV infection is associated with increased circulating levels of IL-7, however memory T-cells are not expanded and homeostasis is not maintained [12], [14], [19]. IL-7 has been proposed for use as an immunotherapeutic agent for the treatment of HIV, but the finding of immunologic failure despite high levels of circulating IL-7 has suggested that this type of therapy may not be beneficial [20], [21]. Dysfunctional CD127 expression [7], [22] suggests that failure of the IL-7/IL-7 receptor system may contribute to the loss of memory T-cell maintenance in HIV infection. As a result, although there is an increase in circulating IL-7, lack of its receptor may result in poor immunological response to IL-7 therapy.

No comprehensive evaluation of CD8+ T-cell expression of CD127 in HIV-infected children has appeared. A recent study of 37 HIV-infected children showed that CD127 on CD8+ T-cells correlated directly with CD4+ T-cell frequency [23]. Our cross-sectional study sought to investigate this correlation, as well as association with other clinical and immunological surrogates of disease severity, in a larger group of HIV-infected children.

## Results

### Study participants

Fifty-one HIV-infected children were enrolled. Three children were excluded from analysis because of technical problems with blood processing; 48 remained in the study. A broad spectrum of HIV disease status and of response to therapy was present among patients (Table 1). Only two children were not receiving antiretroviral therapy; both had CD4+ lymphocyte counts in the normal range and were asymptomatic. Given the broad use of combination antiretroviral therapy (cART) in our patient population, a majority of our study participants had CD4+ T-cell frequencies in the normal or moderately immunosuppressed range.

**Table 1:** Baseline characteristics of 48 HIV-infected children enrolled into the study.

**Age in months (median, range)**	147 (29–241)	
**Male/female ratio**	0.5	
**HIV immunologic stage (n, %)**		
1 (normal CD4 lymphocyte count)	14 (29%)	
2 (moderately immunosuppressed)	12 (25%)	
3 (severely immunosuppressed)	22 (46%)	
Current CD4+ T cell frequency (n, %)		
≥ 25% (normal)	34 (71%)	
15–24% (moderately immunosuppressed)	8 (16%)	
<15% (severely immunosuppressed)	6 (13%)	
**Current viral load (n, %)**		
Undetectable	16 (33%)	
Up to 10,000 copies/mL	20 (42%)	
>10,000 copies/mL	12 (25%)	
**Participants receiving antiretroviral therapy (n, %)**	46 (96%)	
**HIV clinical stage (n, %)**	**Given**	**Current**
N (asymptomatic)	6 (12%)	22 (46%)
A (mildly symptomatic)	11 (22%)	7 (15%)
B (moderately symptomatic)	15 (31%)	6 (12%)
C (AIDS)	16 (33%)	13 (27%)

“HIV immunologic stage” refers to the CDC immunologic classification of the patient, which reflects their nadir CD4+ T cell frequency, and may be different from their “current CD4+ T cell frequency”. “Given” HIV clinical stage refers to participants' CDC classification of symptomatic disease [24]. “Current” HIV clinical stage reflected application of the CDC classification to symptomatic disease at the time of study enrollment. The latter classification was useful because “given” HIV clinical disease stage often reflected previous, severe disease, while many participants on HAART were clinically well at the time of enrollment.

### Relationship of CD127 expression on CD8+ T-cells with immunological HIV disease status

**Figure 1:**
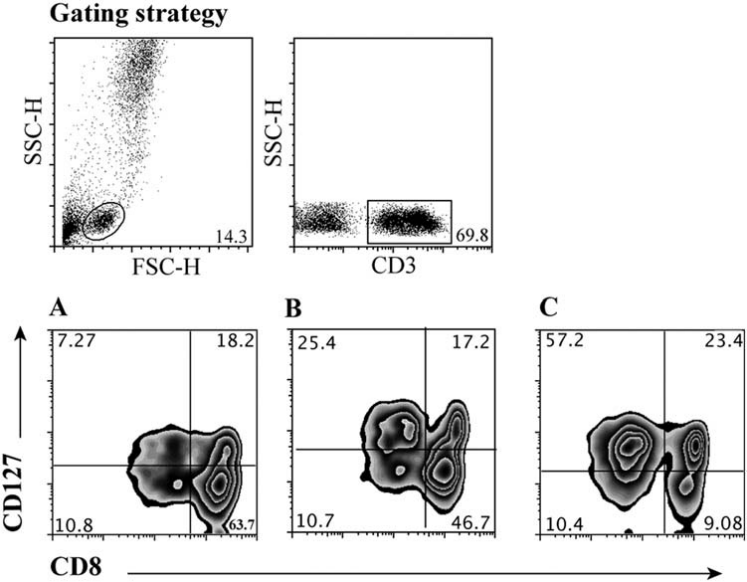
Gating strategy for the identification of CD127 expression on CD8+ T cells. Lymphocytes were selected in a forward scatter height (FSC-H) versus side scatter height (SSC-H) plot. T cells were then selected by gating on CD3+ cells. A–C. Pattern of CD127 expression on CD8+ T-cells in the peripheral blood of 3 of our HIV-infected children with differing CD4+ T-cell frequencies. Results from 3 individual children are shown, with respective CD8+ lymphocyte frequencies of 3% (A), 23% (B) and 46% (C). The values in brackets represent CD127 expression as a frequency of total CD8+ T cells. Results shown are gated on CD3+ T cells.

**Figure 2:**
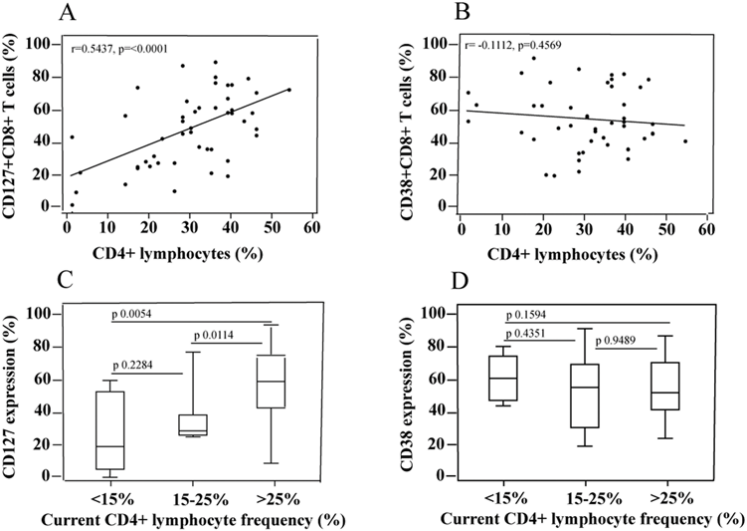
A–B. Association between CD8+ T-cell expression of CD127 (A.) and of CD38 (B.) and CD4+ lymphocyte frequency, in 48 HIV-infected children. A Spearman test was used to assess correlations. C–D. Relationship of CD8+ T-cell expression of CD127 (C) and of CD38 (D) with degree of immunosuppression, as categorized by current CD4+ lymphocyte frequency. Mann Whitney test was used to assess differences between the groups.

Our hypothesis was that IL-7Rα (CD127) expression on CD8+ T-cells (Figure 1) would be a marker of clinical HIV disease status in infected infants, children and adolescents. We first evaluated the relationship between CD127 expression and CD4+ T-cell count, the most widely used immunological surrogate of HIV disease. We confirmed previous observations that there was a strong correlation between CD8+ T-cell expression of CD127 and current CD4+ lymphocyte frequency (Figure 2a) [23]. This association was much stronger than the expected negative association between CD4+ lymphocyte frequency and CD38 expression on CD8+ T cells (Figure 2b), a marker which has been proposed to be a good surrogate of HIV disease status [24].

Similarly, there was a striking association between mean CD127 expression on CD8+ T-cells and current immunologic status categorized as normal, moderately immunosuppressed or severely immunosuppressed, according to current CD4+ lymphocyte frequency (Figure 2c); this association was not present for CD38 expression of CD8+ T cells (Figure 2d).

There was no association between CD127 expression on CD8+ T-cells and CDC “given” immunological classification (data not shown). This classification reflects the nadir of the CD4+ T cell count in infants and does not take into account immune reconstitution that may follow cART.

We concluded that CD127 expression on CD8+ T-cells may be a more reliable marker of HIV-induced immunosuppression than CD38+ expression on these cells. Our results further suggested that CD127 expression on CD8+ T-cells may be a useful indicator of current HIV disease status, as this marker was a reflection of current immunological status.

### Relationship of CD127 expression on CD8+ T-cells with HIV viral load

**Figure 3 pone-0003986-g003:**
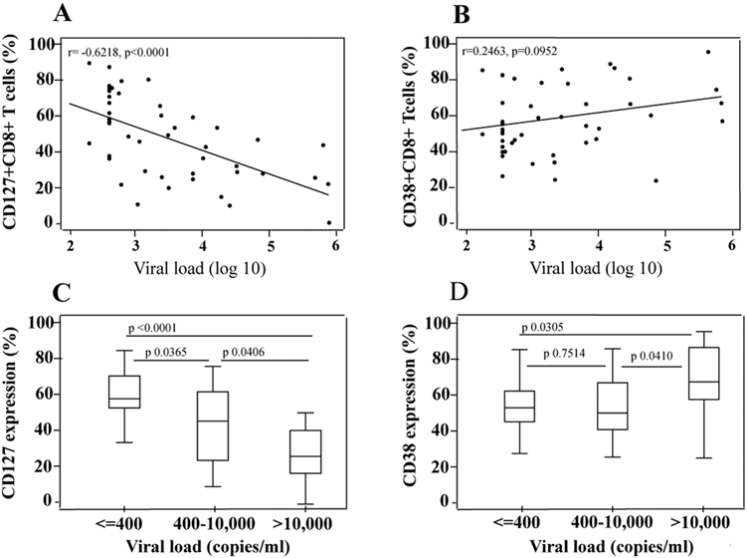
A–B. Association between CD8+ T-cell expression of CD127 (A.) and of CD38 (B.) and plasma HIV viral load, in 48 HIV-infected children. The lower limit of detection of the plasma HIV assay was 400 copies/mL. A Spearman test was used to assess correlations. C.–D. Relationship of CD8+ T-cell expression of CD127(C) and of CD38 (D) with degree of virologic control, as categorized by the plasma HIV viral load. Mann Whitney test was used to assess differences between the groups.

There was a strong negative correlation between CD127 expression on CD8+ T-cells and HIV viral load (Figure 3a), but CD38 expression on these cells was not associated with viral load (Figure 3b). CD127 expression was highest in those children with excellent virologic control (Figure 3c) and lowest in those children with poor virologic control. No differential pattern could be seen for CD38 expression (Figure 3d).

Given these findings, we concluded that the close association of CD127 expression on CD8+ T-cells with HIV viral load further suggested that this marker may be an immunological surrogate of HIV disease status.

### Relationship of CD127 expression on CD8+ T-cells with clinical HIV disease status

**Figure 4:**
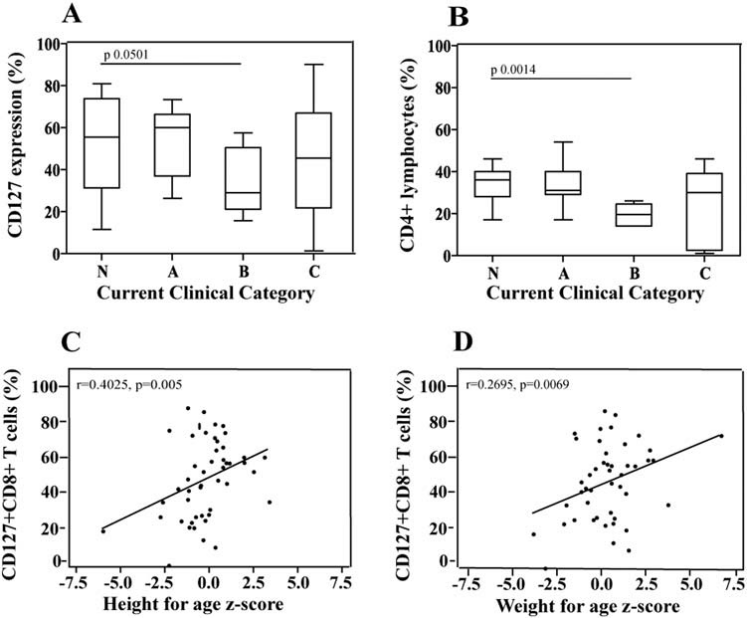
A–B. Relationship between CD8+ T-cell expression of CD127 (A), and of CD4+ T-cell frequency (B), with “current” CDC clinical disease category, in 48 HIV-infected children. ”Current” HIV clinical stage refers to a revised CDC classification, based on symptomatic disease at the time of study enrollment. Mann Whitney test was used to assess differences between the groups. C.–D. Association between height for age z-score (C) and weight for age z-score (D) and CD8+ T-cell expression of CD127. A Spearman test was used to assess correlations.

There was no association between CD127 expression on CD8+ T-cells and “current” CDC classification (Figure 4a). We used current clinical status, and not each patient's given CDC classification, which reflects disease history, since many participants were clinically healthier than the given CDC category would indicate. In this patient population CD38 expression on CD8+ T cells was also not associated with current clinical disease status (data not shown), but neither was the best immunologic surrogate available, CD4+ lymphocyte frequency, associated with clinical disease status (Figure 4b).

Growth may be one of the best indicators of clinical HIV disease status in children [25], and correlates well with immunological and virological parameters to characterize disease status in HIV-infected children [26]. We were able to demonstrate that CD127 expression on CD8+ T-cells was higher when height z scores and weight z scores were higher in our patient population (Figure 4c–d). We did not see a similar association with CD38 expression (data not shown).

This association with growth parameters further strengthened the clinical utility of CD127 expression on CD8+ T-cells as a potential indicator of HIV disease status.

### Multivariate analysis of continuous variables that correlated with CD127 expression on CD8 T-cells

When CD4 T-cell frequency, viral load, weight for age z score, and height for age z-score were assessed in a multivariate model, viral load was the only variable that remained negatively correlated with CD127 expression on CD8+ T-cells (p = 0.011). The bivariate correlation between CD127 expression and the other 3 variables disappeared (p = 0.340, 0.11 and 0.078 for CD4 T-cell frequency, weight for age z-score and height for age z-score, respectively). The correlation between CD4 T-cell frequency and CD127 expression disappeared due to the former variable's strong correlation with viral load and anthropometric outcomes.

We concluded that there was an independent, strong association between plasma viral load and CD127 expression on CD8+ T-cells.

## Discussion

We showed, in a cross-sectional cohort of HIV-infected children, that CD127 expression on CD8+ T-cells is lower when CD4+ T-cell counts are lower, HIV viral loads are higher, and weight and height for age z scores are lower, all indicators of poor control of HIV disease. These associations are compelling and suggest that CD127 expression on CD8+ T-cells could be used along with CD4+ T-cell counts and HIV viral load as a potential indicator of disease status in HIV-infected children.

Our results confirmed and expanded on previous observations in HIV-infected individuals, which have suggested that loss of CD127 expression on CD8+ T-cells may be a correlate of disease progression [7]–[15]. These earlier studies showed that lower CD127 expression occurred both on naïve and memory T-cells in HIV infection, and that there was a strong association with plasma viremia and CD4+ T-cell depletion. Additionally, there was a relationship between low CD127 expression and other T-cell markers suggestive of poor prognosis. These included markers of immune activation, such as increased Ki-67 and HLA-DR expression, and markers suggesting pro-apoptotic activity, such as increased expression of CD95 and decreased expression of Bcl-2 [7]–[15].

Longitudinal studies of HIV-infected children are required to determine whether CD127 expression indeed has utility as another indicator of HIV disease status or disease progression. These studies may also be used to determine whether recovery of expression will occur in response to therapy, as has been suggested in HIV-infected adults, and to assess whether, and in what clinical situations, CD127 expression on CD8+ T-cells may be used to guide antiretroviral therapy. We hypothesize that CD127 expression on CD8+ T-cells may help to guide clinical management, particularly when CD4+ T-cell count and viral load findings are discrepant. The promise of CD127 expression on CD8+ T cells as an immunological correlate was shown when we compared expression of this marker with that of CD38 on CD8+ T cells, a more established marker [24], [27], [28]–CD127 expression was invariably correlated better with HIV viral load and CD4+ T cell counts.

CD127 is preferentially expressed on central memory CD4+ and CD8+ T cells, and is progressively lost with advancing HIV disease [29]–[31]. Similarly, poor virologic control in chronic hepatitis B and C infection is associated with loss of this receptor [32], [33]. The ligand for CD127, IL-7, is, together with IL-15, critical for CD8+ T-cell memory generation and maintenance [16]–[18], [34]. Therefore, both IL-7 and IL-15 therapy have been proposed as immunomodulatory therapies in HIV-infected persons [16], [35]–[37]. Adults with HIV infection already have high plasma IL-7, and these levels appear to correlate inversely with CD127 expressed on T-cells [12]–[14]. It is proposed that, because IL-7 secretion is relatively constant, serum levels of this cytokine are determined by availability of target T-cells expressing CD127 [16]. The consistent observation that CD127 expression decreases with advancing HIV disease therefore raises concerns for IL-7 immunotherapy. Antiretroviral therapy may reverse CD127 expression partially [9], suggesting that IL-7 therapy may be effective in the presence of this intervention. We hypothesize that even in this setting measurement of CD127 expression on CD8+ T-cells may prove to be clinically useful marker for predicting responsiveness to IL-7 administration.

First results of recombinant IL-7 therapy as immunomodulation in persons with refractory cancer have recently been published [38]. The results indicate that therapy could enhance and broaden immune responses, particularly in individuals with limited naive T cells and diminished TCR repertoire diversity, as occurs in HIV infection.

## Methods

### Patient enrollment

In a cross-sectional evaluation, HIV-infected patients were enrolled from the pediatric HIV clinic of the University of Miami Miller School of Medicine. Children with any acute illness or with anemia (hemoglobin≤10 g/dL) were excluded. At enrollment, medical information, including HIV viral loads, CD4+ T-cell counts and treatment with cART, was obtained from the participant's medical record. A physical examination was performed, including height and weight measurements. The protocol for this study was approved by the Institutional Review Board of the University of Miami. Ethical guidelines of the United States Department of Health and Human Services were followed, which included written informed parental consent from parents or guardians, and assent from participants≥7 years of age.

### Blood collection and processing

Sodium heparinized blood was collected, and FACS Lysing Solution (BD Biosciences) was added to lyse red cells and fix white cells. These samples were stored overnight at 4°C, prior to staining for flow cytometric analysis of T-cell subsets the next day. Preliminary studies demonstrated that this cellular processing yielded flow cytometry results that were identical to those obtained when fresh, unfixed cells were used. As part of the clinical patient visit, blood was collected simultaneously for HIV viral load testing (Amplicor v1.5, Roche, either standard or ultrasensitive at the discretion of the clinical provider), clinical CD4+ T cell count, and complete blood count.

### Antibodies

Fluorescence-conjugated antibodies against CD45, CD3, CD8, and CD4 were obtained from BD Biosciences, while a fluorescence-conjugated antibody against IL-7Rα (CD127) was obtained from Beckman-Coulter (clone R34.34).

### Flow cytometric analysis of T-cell subsets

Lymphocytes obtained after incubation of the whole blood with FACS Lysing Solution were pelleted, washed, and stained with combinations of fluorescence-conjugated antibodies. Two panels were used to examine cells: (1) CD45, CD3, CD4 and CD8, and (2) CD3, CD38, CD8, CD127. Acquisition was performed with a FACS Calibur flow cytometer (BD Biosciences), and further analysis was completed with FlowJo software (Treestar). In the first panel, CD4+ and CD8+ lymphocyte and T-cell frequencies were determined after gating on CD45+ lymphocytes. For the second panel, frequency of CD127 expression and CD38 expression on CD8+ T-cells, defined as CD3+ plus CD8+, were measured. CD127 percentages reflected in figures 2–4 were calculated as a frequency of the total CD8+ T cell population.

### Statistical considerations

All continuous variables that were not normally distributed were log transformed for analysis. Associations between continuous measurements were illustrated using two-way scatter plots and quantified using Spearman's correlation coefficients. Median values of continuous responses for different groups of patients were compared using the Mann-Whitney test. Linear regression analysis was used to explore predictors of CD127 in a multivariate setting. Different models were compared using likelihood ratio chi-square statistics. The participants were evaluated on the basis of both their “given” and “current” CDC classification. “Given” HIV clinical stage referred to participants' CDC classification of symptomatic disease[39]. We assigned the participants a “current” HIV clinical stage based on applying the CDC classification to the patient's current symptoms at the time of study enrollment. The latter classification was useful because “given” HIV clinical disease stage often reflected previous, severe disease, while many participants on HAART were clinically well at the time of enrollment. For example, a patient with a history of an opportunistic infection at a prior to receiving HAART may have a “given” CDC clinical category C, but may currently have improved clinical status and could be considered category A. Similarly, given that many patients have had immune reconstitution due to HAART, their “current” immunologic category may also be different from their “given” CDC classification.
